# Analysis of *Escherichia coli *O157 clinical isolates by multilocus sequence typing

**DOI:** 10.1186/1756-0500-3-343

**Published:** 2010-12-21

**Authors:** Swaraj Rajkhowa, Joy Scaria, Daniel L Garcia, Kimberlee A Musser, Bruce L Akey, Yung-Fu Chang

**Affiliations:** 1 Department of Population Medicine and Diagnostic Sciences, College of Veterinary Medicine, Cornell University, Ithaca, New York 14853, USA; 2 New York State Department of Health, Wadsworth Center-Axelrod InstituteAlbany, New York 12208, USA

## Abstract

**Background:**

Although many strain typing methods exist for pathogenic *Escherichia coli*, most have drawbacks in terms of resolving power, interpretability, or scalability. For this reason, multilocus sequence typing (MLST) is an appealing alternative especially when applied to the typing of temporal and spatially separated isolates. This method relies on an unambiguous DNA sequence analysis of nucleotide polymorphisms in housekeeping genes and has shown a high degree of intraspecies discriminatory power for bacterial and fungal pathogens.

**Results:**

Here we used the MLST method to study the genetic diversity among *E. coli *O157 isolates collected from humans from two different locations of USA over a period of several years (2000-2008). MLST analysis of 33 *E. coli *O157 patient isolates using the eBurst algorithm distinguished 26 different sequence types (STs), which were clustered into two clonal groups and 11 singletons. The predominant ST was ST2, which consisted of 5 isolates (14.28%) followed by ST1 (11.42%). All the isolates under clonal group I exhibited a virtually similar virulence profile except for two strains, which tested negative for the presence of *stx *genes. The isolates that were assigned to clonal group II in addition to the 11 singletons were found to be phylogenetically distant from clonal group I. Furthermore, we observed a positive correlation between the virulence profile of the isolates and their clonal origin.

**Conclusions:**

Our data suggests the presence of genetic diversity among *E. coli *O157 isolates from humans shows no measurable correlation to the geographic origin of the isolates.

## Background

Shiga toxin-producing *E. coli *(STEC) O157 has emerged as a public health threat following its initial identification as a pathogen in a 1982 outbreak of illness associated with the consumption of undercooked ground beef [1]. Specifically, *E. coli *O157:H7 and O157:NM (nonmotile) are recognized as major etiologic agents in hemorrhagic colitis (HC) and hemolytic-uremic syndrome (HUS) in humans. This enteric organism is able to secrete Shiga toxin, a binary toxin that affects the endothelium of the kidney, gut and brain that can result in glomerular vascular damage, bloody diarrhea, and brain edema, distinguishing it from other pathogenic strains of *E. coli*. STEC has been implicated as the source of numerous outbreaks and sporadic cases stemming predominantly from consumption of bovine food products. Transmission has also been linked to unpasteurized milk and cider, contaminated drinking and swimming water, fresh vegetables and secondarily through person-to-person contact. Infection due to *E. coli *O157:H7 or enterohemorrhagic *E. coli *(EHEC) is typically characterized by diarrhea, abdominal cramping and hemorrhagic colitis. Hemolytic uremic syndrome and thrombotic thrombocytopenic purpura are less common, but severe sequelae of infection.

The public health impact of EHEC infections is high because of systemic complications from infections, such as HUS (an important cause of acute renal failure in childhood), late post-infection sequelae[2] and the ability of STEC to cause large outbreaks. To date, EHEC O157:H7 has caused hundreds of outbreaks worldwide[3]. The largest known outbreak on record occurred in Japan, in Sakai City, in 1996[4], where thousands, mostly school children, were affected. The U.S. Centers for Disease Control and Prevention estimates that *E. coli *O157:H7 causes approximately 73,400 illnesses and 60 deaths each year in the United States[5]. Cattle are a major reservoir of *E. coli *O157:H7[6], but additional potential reservoirs of this pathogen include sheep, goats, pigs, horses, dogs, poultry, and deer[7-10].

The ability to identify accurately and track the strains of infectious agents that cause disease is central to epidemiological surveillance and public health decisions, but there are no wholly satisfactory methods of achieving this goal[11]. All of the numerous methods that are currently used suffer from one or more significant drawbacks, including inadequate discrimination, limited availability of reagents, poor reproducibility within and between laboratories, and ability to quantitatively define genetic relationships between isolates. However, perhaps the most important limitation of current typing methods is the difficulty of comparing the results achieved by different laboratories.

Molecular typing methods are used to address two very different kinds of problems. First, for short-term or local epidemiology, the isolates recovered from a localized outbreak of disease need to be accurately grouped using previously characterized isolates as benchmarks. Second, for long-term or global epidemiology, pathogenic strains collected over relatively long time periods and/or from disparate geographic regions must be correctly classified as related or not to those isolated world-wide. Different methods may be appropriate for investigating local and global epidemiology, but in both cases they should be discriminatory enough so that isolates can be classified.

High specificity levels of isolate strain identity can be achieved in two different ways. In one approach, individual loci, or uncharacterized and dispersed regions of the genome, that are highly variable within the bacterial population are identified. For bacterial pathogens, several methods based on this approach are currently popular, e.g., ribotyping, pulsed-field gel electrophoresis (PFGE), and PCR with repetitive element primers, or arbitrary primers [11]. For these methods, restriction enzymes (or PCR primers) are chosen that yield maximal variation within the population; consequently, the variation that is indexed is evolving very rapidly, usually for reasons that are not clear. The second approach, typified by multilocus enzyme electrophoresis (MLEE), is to use variation that accumulates very slowly in the population and is likely to be selectively neutral. Although only a small number of alleles can be identified within the population by using this type of variation, high levels of discrimination are achieved by analyzing multiple loci.

Methods that index rapidly evolving variations are useful for short term epidemiology but may generate misleading results when applied in a global epidemiological survey. Several studies have shown that techniques such as PFGE resolve isolates that are indistinguishable by MLEE. For example, MLEE studies of populations of *Salmonella enterica *have shown that isolates of serovar Typhi from typhoid fever belong to one of two closely related electrophoretic types (ETs)[12]. In contrast, isolates of serovar Typhi are relatively diverse according to PFGE[13]. PFGE is therefore useful for studying individual outbreaks of typhoid fever because, unlike MLEE, it identifies the microvariation (some of which is caused by transient or unstable genetic elements or regions i.e. phages, transposons, genetic rearrangements, repetitive elements etc) that is needed to distinguish between strains circulating within a given geographic area. However, this technique is not well suited for long term epidemiology (and occasionally for short term epidemiology for reasons described above) because it does not indicate that isolates that cause typhoid fever are members of a single globally distributed clonal lineage of *S. enterica*. Metaphorically, PFGE and other similar methods may cause epidemiologists not to see the forest for the trees.

The best current techniques for long term epidemiology, and for the identification of lineages that have an increased propensity to cause disease, is undoubtedly MLEE. This approach also has contributed greatly to our understanding of the global epidemiology and population structure of infectious agents. For many pathogens, MLEE successfully has identified clusters of closely related strains (clones or clonal complexes) that are particularly liable to cause disease[11]. However, a major problem with MLEE, and virtually all other current (gel-based) typing methods, is that the results obtained in different laboratories are difficult to compare.

Multilocus sequence typing (MLST) uses nucleotide sequences of internal fragments of selected genes as the unit of comparison and, therefore, does not suffer from the drawbacks of gel-based fingerprinting methods. Sequence data are unambiguous, more easily comparable, and transferable between laboratories and are highly reproducible[11]. Furthermore, the digital format of MLST data has facilitated the establishment of global, web-accessible databases for a variety of organisms and is rapidly contributing to our understanding of the clonal distribution of infectious disease agents. The most commonly used MLST schemes index the nearly neutral genetic variation in housekeeping genes, which are believed to evolve slowly because they are for the most part, under stabilizing, selective pressure. MLST is thus a powerful tool for global and long-term surveillance.

Various authors [13-16] have reported the use of MLST methodology for the study of *E. coli*, particularly the serogroup O157. These authors have reported use of various house keeping genes for analysis of *E. coli *O157 strains by MLST. The development of an effective MLST scheme for subtyping *E. coli *O157:H7 has been hindered in the past due to lack of sequence variation found within analyzed housekeeping and virulence genes. A recent study suggested that *rhs *genes are under strong positive selection pressure and therefore, may be useful markers for phylogenetic analysis of *E. coli *O157:H7 [15-17]. EHEC differ from EPEC in that they produce Shiga toxins but not bundle-forming pili. The same authors also reported that *E. coli *with the O157 O antigen are not always EHEC but may belong to other pathotypes. They also described two serogroups of O157 *E. coli *strains from Brazilian infants with diarrhea and shown with variety of assays that those strains belonged to the enteropathogenic, not the enterohaemorrhagic pathotype. The putative virulence factors of O157:H7 strains, the ability to produce Shiga-like toxins and adhere to epithelial cells, also exist in other groups of *E. coli *[18].

In this study we have performed MLST on a set of *E. coli *O157 clinical isolates obtained from two different locations of USA to determine whether (or not) any genetic diversity exists among the isolates.

## Results

### Multilocus sequence analysis

Nucleotide sequence analysis of unlinked housekeeping genes (multilocus sequence typing) is widely used for evolutionary and population analysis, and also for epidemiological investigations. Since differences in the sequences of essential housekeeping genes are thought to display long-term genetic changes, we used this technique to investigate the phylogenetic relationships of *E. coli *O157 clinical isolates obtained from New York State and Pennsylvania State of USA. DNA sequences of corresponding housekeeping genes from the O157:H7 strains EDL933 (accession no. NC_002655) and Sakai (accession no. NC_002695) were compiled from the respective published genome sequences and used as reference strains for this study. Sequence data from each isolate, including the two published strains, was then garnered from each of the nine loci. Forward and reverse sequence reads from each locus were aligned and edited.

Analogous to other MLST schemes, we assigned the allele numbers to each unique allele sequence for each of the nine loci investigated. The number of alleles identified for the 33 *E. coli *O157 isolates varied from 3 to 14 per gene; *ompA *generated the least number of alleles (3 alleles), while *pgm *generated the most (15 alleles). The combinations of allele numbers for all isolates are shown in Table 1. Each unique combination of allele numbers represents one sequence type (profile). The allelic diversity found at the nine loci resulted in 26 unique ST for the 33 isolates. Five (14.28%) of these showed MLST profile 2, four isolates (11.42%) showed MLST profile 1 and the remaining 24 strains fell into MLST profile 3 to 26.

**Table 1:** Allele frequencies of nine MLST genes in E. coli O157 isolates from this study

Allele	*arcA*	*aroE*	*dnaE*	*gapA*	*gnd*	*mdh*	*pgm*	*espA*	*ompA*
1	21	26	23	29	26	25	6	27	31
2	1	1	1	2	1	1	10	1	1
3	1	1	1	1	3	1	1	2	1
4	4	5	3	1	2	1	1	1	-
5	1	-	1	-	1	5	2	2	-
6	1	-	1	-	-	-	1	-	-
7	1	-	2	-	-	-	1	-	-
8	1	-	1	-	-	-	2	-	-
9	1	-	-	-	-	-	2	-	-
10	1	-	-	-	-	-	1	-	-
11	-	-	-	-	-	-	2	-	-
12	-	-	-	-	-	-	2	-	-
13	-	-	-	-	-	-	1	-	-
14	-	-	-	-	-	-	1	-	-
Total	10	4	8	4	5	5	14	5	3

**Figure 1:**
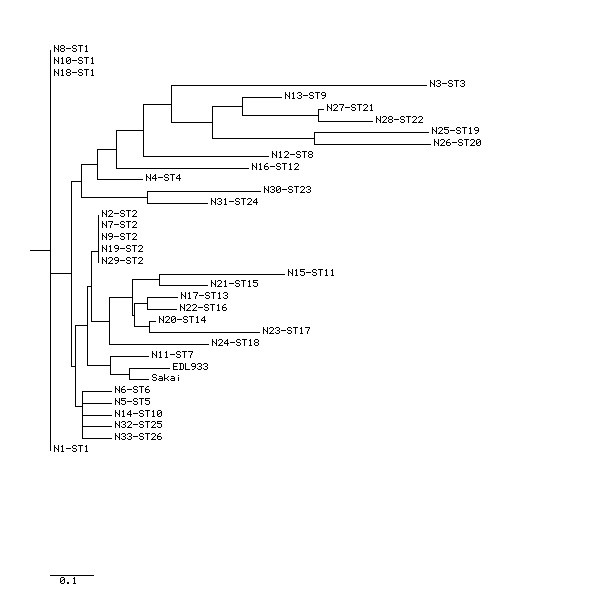
**Dendrogram (based on Neighbor Joining Method) showing the genetic relatedness among the 33 *E. coli *O157 isolates**. The genetic relatedness of the isolates with the reference strains EDL933 Sakai are also showed in the figure. The isolate number and corresponding sequence type is also shown.

Figure 1 shows a dendrogram drawn by using Tree drawing, a web-based software used for displaying phylogenies in the form of tree dendrograms. From the dendrogram it has been observed that the isolates in 12 profiles (ST1, ST2, ST5, ST6, ST7, ST10, ST13, ST14, ST15, ST16, ST25 and ST26) are found to be closely related to the reference strains EDL933 and Sakai in comparison to rest of the isolates in the study. Isolates no. 27 and 28 (ST21 and ST22) are also closely related to each other.

**Figure 2:**
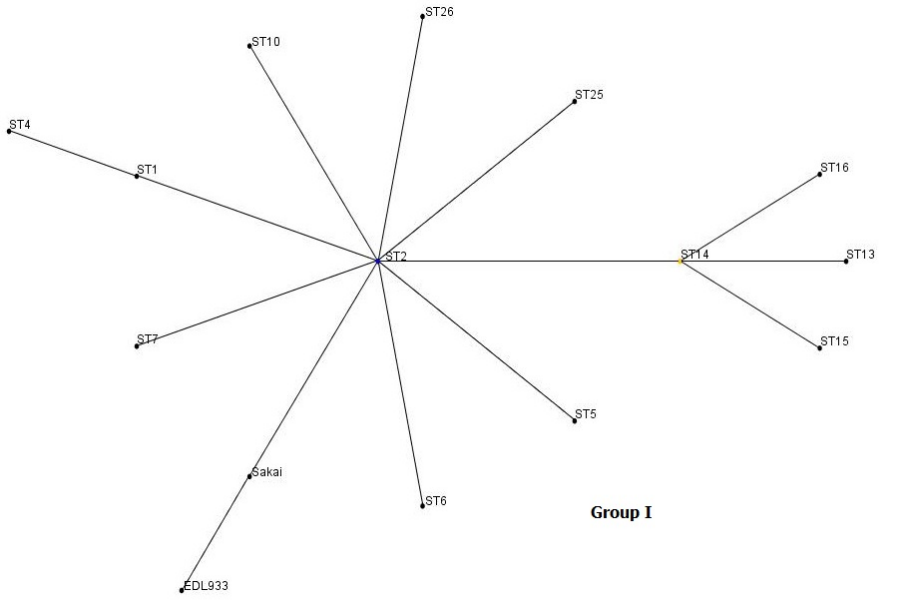
**Clonal groupings among *E. coli *O57 isolates: Allelic profiles were analyzed by eBurst and groups were defined as sets of related isolates sharing identical alleles at eight of nine loci with at least one other member of the group**. Blue dot in group I indicates putative founder, yellow dot that of subgroup.

**Figure 3:**
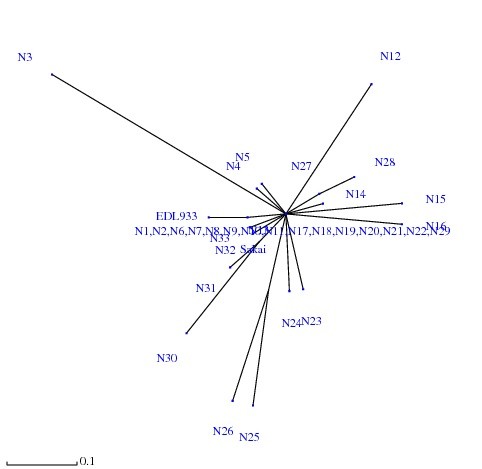
**SplitsTree analysis showing the genetic relatedness among the 33 *E. coli *O157 Isolates**. The genetic relatedness of the isolates with the reference strains EDL933 Sakai are also showed in the figure.

Analysis by eBurst revealed two non-overlapping groups or clonal complexes, consisting of related isolates sharing identical alleles at eight out of the nine loci with at least one other member of the group (Figure 2). The relatedness among the 33 *E. coli *O157 isolates was also analyzed with SplitsTree, an alternative algorithm for the analysis and visualization of evolutionary data that is not always best represented by a standard tree. The SplitsTree graph (Figure 3) showed a clustering of strains highly similar to those obtained with both the eBurst algorithm and dendrogram (based on Neighbor Joining Method).

### Analysis of virulence markers

All *E. coli *O157 isolates were investigated for the presence of virulence genes associated with EHEC (Table 2). All isolates were also screened for the presence of *bfpA *gene (which is associated with typical EPEC), because of the growing evidence that some EHEC share common genes with EPEC. All isolates except for two (isolate no. N6 and N9) were positive for at least one of the Shiga toxin genes (either *stx1 *or *stx2 *or combination of both). *eaeA *and *hlyA *genes were carried by 78.78% and 60.60% of the isolates, respectively. None of the isolates were found to be positive for *bfpA*.

**Table 2:** PCR primers used for amplification of virulence genes

Target gene	Primer designation	Primer sequence (5'-3')	Amplicon size (bp)	Reference
*stx1*	stx1F	ATAAATCGCCATTCGTTGACTAC	180	(26)
	stx1R	AGAACGCCCACTGAGATCATC		
*stx2*	stx2F	GGCACTGTCTGAAACTGCTCC	255	
	stx2R	TCGCCAGTTATCTGACATTCTG		
*eaeA*	eaeAF	GACCCGGCACAAGCATAAGC	384	
	eaeAR	CCACCTGCAGCAACAAGAGG		
*hlyA*	hlyAF	GCATCATCAAGCGTACGTTCC	534	
	hlyAR	AATGAGCCAAGCTGGTTAAGCT		
*bfpA*	bfpA	AATGGTGCTTGCGCTTGCTGC	326	(9)
	bfpR	GCCGCTTTATCCAACCTGGTA		

## Discussion

Molecular epidemiology is largely descriptive and characterizes bacteria based on their natural genetic variation. A number of molecular methods have been employed to determine this variation, which may be due to mutation or horizontal gene transfer events. Basically two branches of molecular epidemiology can be distinguished, classification and typing. Classification can illustrate evolutionary relationships and groups species into clonal groups or complexes, whereas typing is used more often for differentiation of clinical or environmental isolates. Long-term epidemiology may require classification, whereas short-term local epidemiology is more often performed by high resolution typing methods. While the most prominent methods for molecular classification are MLST and multilocus enzyme electrophoresis[11], PFGE or PCR-based methods such as randomly amplified polymorphic DNA-PCR, AFLP or virulence gene characterization, have been used frequently for typing approaches[19]. In our study, we used MLST in combination with PCR detection of selective virulence genes for classification of pathotypes and for phylogenetic analysis of *E. coli *O157 isolates. The isolates were collected from patients over a period of several years (2000-2008) from two different states of the United States of America., Some of these isolates were associated with outbreak cases.

In our study, MLST analysis of nine genes (seven housekeeping genes and two membrane protein coding genes) revealed 26 profiles from 33 *E. coli *O157 isolates. Analysis by eBurst revealed two clonal groups or clonal complexes (clonal group I and II). All the isolates in clonal group I were closely related to reference strains EDL933 and Sakai. It has also been observed that the leading markers of clonal group I are the presence of *stx1 *and/or *stx2 *genes, *eaeA *gene and *hlyA *gene, characterizing them as typical STEC. The only difference was that the isolate N29 and N32 were negative for the presence of the *hlyA *and *eaeA *genes, respectively. We also observed that, of the nine loci investigated, the *pgm *gene generated the highest number of alleles, which could be due to use of a larger DNA fragment for amplification of the *pgm *gene in the study.

One surprising finding of our study is that although the isolates N6 and N9 were negative for stx genes still they belonged to the same clonal group along with the EDL933 and Sakai strains. It is possible that these strains might have lost the phage-borne *stx *genes upon subculture as reported for other STEC[20,21]. Spontaneous loss of both *stx1 *and *stx2 *genes *in vitro *has also been described in *E. coli *O157:H7 clinical isolate[22]. The timing of the stool sample collection is also very important for finding Stx producing strains in patients with HUS. Tarr et al. demonstrated that if the stool samples of patients with HUS were cultured within 6 days after onset of diarrhea for EHEC O157:H7, the recovery rate was nearly 100% [23]. This rate decreased to 33.3% in stool samples collected >6 days after the onset of diarrhea. Mellmann et al. also demonstrated that EHEC are difficult to identify in patient's feces at late stages in illness[24].

Friedrich et al. has proposed two possible explanations for the findings of *stx*-negative *E. coli *O157 strains related to sporadic cases of diarrhea and HUS and to outbreaks[25]. First, *stx*-negative *E. coli *O157 strains are thought to have evolved from *stx*-positive *E. coli *O157 of an original infection by loss of *stx *genes during the course of infection. Alternatively, *E. coli *O157 strains that are inherently *stx*-negative from the beginning might be responsible for the disease in these cases.

Maintaining the Stx-encoding phage may adversely affect or be lethal to the bacterium when changes occur in normal environmental conditions or may become so when in the human host during the course of infection. Survival might be favored by loss of the phage, since *stx*^- ^progeny of *stx*^+ ^progenitors are less prone to lysis. Mammalian host signals such as those initiated by exposure to hydrogen, can perhaps induce Stx-encoding prophages to become lytic[26]. By generating *stx*^- ^mutants, a strain can survive without automatically lysing (because it no longer carries the lysogenic phage or the phage-encoded toxin). The loss of *stx*-encoding phage can thus offer a selective advantage.

The finding of *stx*-negative *E. coli *O157 isolates has clinical significance and is important from a diagnostic standpoint because in such cases, *stx*- and Stx-independent procedures are required to detect strains that might have lost their *stx *genes. Our study indicates that routine testing of stools for either Stx by ELISA or the *stx *genes by PCR or merely relying on the results from culturing *E. coli *O157 on sorbitol MacConkey agar, may not identify all potentially important factors that are associated with diarrhea and HUS. The same observation was made during recent outbreaks of gastroenteritis in the United States. Recently Bielaszewska et al.[24] also reported that at the time of microbiological analysis, ~5% of HUS patients no longer shed the causative EHEC, but excrete *stx*-negative derivatives of EHEC that have lost *stx *during the course of infection. In such patients, the EHEC etiology of HUS is missed using current methods, which rely solely on detecting *stx *or Shiga toxin which can hamper epidemiological investigations and lead to inappropriate clinical management. Therefore, it is suggested that Stx- and *stx *gene-based detection methods should be complemented by additional methods for the identification of stx-negative *E. coli *O157 in microbiologic evaluations.

We observed in our study that the clonal group II consisted of only two isolates (N27 and N28) both of which share a similar virulence profile yet their virulence profile was different from the majority of the isolates under clonal group I. We have also observed that 11 isolates did not belong to any clonal group and instead, each isolate exhibited a different sequence type (profile). These isolates are only distantly related phylogenetically to clonal group I. The reasons for this can not be explained with MLST data alone; this points to towards the need of whole genome sequencing of larger number O157 strains. However, our present findings are in agreement with the findings of Ogura et al. who observed a high level of genetic diversity among *E. coli *O157 strains from human isolates[27]. The greater genetic diversity observed among *E. coli *O157 isolates in our study could be due to several factors like (i) inter-species transfer of strains to humans from farm animals/pets, (ii) contaminated food sources (iii) increased international travel and (iv) longer span of sample collection period (2000-2008).

## Conclusions

Our study shows that all the methods compared here (dendrogram based on Neighbor Joining Method, eBurst algorithm and SplitsTree) exhibited a high degree of concordance showing a highly similar clustering pattern for the strains thereby indicating that the sequence data were reliable and were accurately represented by these algorithms. Infections with EHEC O157 may involve asymptomatic carriage or uncomplicated diarrhea, but other outcomes include haemorrhagic colitis and HUS. This poses considerable challenges both clinically and for disease control measures, because the disease is not yet treatable and colonization of humans can occur with only a few organisms. Although there are reports on clonal diversity and pathogenic properties of *E. coli *O157 strains, the present study extends our knowledge about the virulence spectrum and genetic relationships of *E. coli *O157 isolates that were collected only from one host (humans) over a period of several years (2000-2008). Phylogenetic analysis has demonstrated genetic diversity among *E. coli *O157 isolates collected from humans and thereby warrants further in-depth studies involving a larger number of samples so that more information about their diversity can be obtained.

## Materials and methods

### Bacterial isolates

A total of 33 *Escherichia coli *O157 isolates (23 from the Wadsworth Center, New York State Department of Health, Albany, New York, USA and 10 from Pennsylvania State, USA) were used in the study. The samples were collected from the patients with HUS, diarrhea or asymptomatic. Age group of the patients varied from 1 year to 42 years. The characteristics of the isolates used are shown in Table 3. Reference strains used in this study were EDL933 (accession no. NC_002655) and Sakai (accession no.NC_002695).

**Table 3:** Properties of E. coli O157 isolates investigated in the study

Strain designation	MLST ST	Clonal group	Origin	Virulence genes				
				
				*stx1*	*stx2*	*eaeA*	*hlyA*	*bfpA*
N1	ST1	I	PNN	+	+	+	+	-

N2	ST2	I	PNN	+	+	+	+	-

N3	ST3	S	NY	+	-	-	-	-

N4	ST4	I	PNN	+	+	+	+	-

N5	ST5	I	PNN	+	+	+	+	-

N6	ST6	I	NY	-	-	+	+	-

N7	ST2	I	NY	+	+	+	+	-

N8	ST1	I	NY	+	+	+	+	-

N9	ST2	I	NY	-	-	+	+	-

N10	ST1	I	PNN	+	+	+	+	-

N11	ST7	I	NY	+	+	+	+	-

N12	ST8	S	NY	+	+	+	-	-

N13	ST9	S	NY	+	+	+	-	-

N14	ST10	I	NY	+	+	+	+	-

N15	ST11	S	NY	+	+	+	-	-

N16	ST12	S	NY	+	+	-	-	-

N17	ST13	I	NY	+	+	+	+	-

N18	ST1	I	NY	+	+	+	+	-

N19	ST2	I	NY	+	+	+	+	-

N20	ST14	I	PNN	+	+	+	+	-

N21	ST15	I	PNN	+	+	+	+	-

N22	ST16	I	PNN	+	+	+	+	-

N23	ST17	S	NY	+	+	+	-	-

N24	ST18	S	NY	+	+	+	-	-

N25	ST19	S	NY	+	-	-	-	-

N26	ST20	S	NY	+	-	-	-	-

N27	ST21	II	NY	+	+	-	-	-

N28	ST22 s ST22	II	NY	+	-	-	-	-

N29	ST2	I	NY	+	+	+	-	-

N30	ST23	S	NY	+	+	+	-	-

N31	ST24	S	NY	+	+	+	+	-

N32	ST25	I	PENN	+	+	-	+	-

N33	ST26	I	PENN	+	+	+	+	-

NY: New York, PENN: Pennsylvania, S: Singleton, + positive, -negative

### Examination of virulence genes

The virulence genes were investigated by PCR as listed in Table 2.

### Selection of genes

The following seven housekeeping genes were included in the study: *arcA *(aerobic respiratory control protein), *aroE *(shikimate dehydrogenase), *dnaE *(DNA polymerase III, alpha subunit), *mdh *(melate dehydrogenase), *gnd *(6-phosphogluconate dehydrogenase), *gapA *(glyceraldehyde 3-phosphate dehydrogenase), and *pgm *(phosphoglucomutase). In addition to these genes, two membrane protein coding genes *espA *(*E. coli *secreting protein A) and *ompA *(outer membrane protein A) were also included. The seven selected housekeeping genes were chosen for their potential sequence diversity. Three of the genes, *aroE, arcA *and *mdh *have been used to determine the evolution of pathogenic bacteria[28]. Two genes, *dnaE *and *pgm *were chosen because they were found to be informative for *Salmonella *and *Vibrio cholerae*[29]. The last two housekeeping genes, *gapA *and *gnd *were chosen because they were transferred into the O157 genome at different evolutionary times. Finally, the two membrane proteins were chosen as being potential targets of the immune system and presumably, under balancing selective pressure. The primers for these genes were from Noller et al[13] except for genes *pgm *and *espA*. The primers for these two genes were designed based on the published sequences from Genbank (Table 4).

**Table 4:** PCR primers and conditions for multilocus sequence analysis

Gene	Primer sequence^a^	Reaction parameters^b^	Amplicon size (bp)	Gene ID	Locus tag
*arcA*	F: 5'-GAAGACGAGTTGGTAACACG-3' R: 5'-CTTCCAGATCACCGCAGAAGC-3'	95°C for 1 min, 55°C for 2 min, 72°C for 3 min, 30 cycles	680	959654	Z6004
*aroE*	F: 5'-AAGGTGCGAATGTGACGGTG-3' R: 5'-AACTGGTTCTACGTCAGGCA-3'	95°C for 1 min, 57°C for 2 min, 72°C for 3 min, 28 cycles	620	961663	Z2720
*dnaE*	F: 5'-GAG/TATGTGTGAGCTGTTTGC-3' R: 5'-CGA/GATA/CACCGCTTTCGCCG-3'	94°C for 45 s, 45°C for 45 s, 72°C for 1 min, 30 cycles	550	956917	Z0196
*mdh*	F: 5'-CAACTGCCTTCAGGTTCAGAA-3' R: 5'-GCGTTCTGGATGCGTTTGGT-3'	94°C for 45 s, 50°C for 45 s, 72°C for 1 min, 30 cycles	580	958670	Z4595
*gnd*	F: 5'-GGCTTTAACTTCATCGGTAC-3' R: 5'-TCGCCGTAGTTCAGATCCCA-3'	94°C for 45 s, 50°C for 45 s, 72°C for 1 min 10 s, 30 cycles	590	962087	Z3191
*gapA*	F: 5'-GATTACATGGCATACATGCTG-3' R: 5'-CAGACGAACGGTCAGGTCAAC-3'	94°C for 45 s, 50°C for 45 s, 72°C for 1 min 10 s, 30 cycles	535	961753	Z2818
*pgm*	F: 5'-CCGTCCCATAACCCGCCGGAAG ATGGTGGTATCAAGTACAATCCG-3' R: 5'-TTACGCGTTTTTCAGAACTTCGCT AACAATCTCAACCGCTTCTTT-3'	94°C for 45 s, 50°C for 45 s, 72°C for 1 min, 35 cycles	1146	957767	Z0837
*espA*	F: 5'-ATGGATACATCAAATGCAACATC CGTTGTTAATGTGAGTGCGAGT-3' R: 5'-TTATTTACCAAGGGATATTGCTG AAATAGTTCTATATTGTAGAGA-3'	94°C for 45 s, 50°C for 45 s, 72°C for 1 min, 35 cycles	579	960865	Z5107
*ompA*	F: 5'-AGACAGCTATCGCGATTGC-3' R: 5'-GCTTTGTTGAAGTTGAACAC-3'	94°C for 45 s, 50°C for 45 s, 72°C for 1 min, 30 cycles	691	958948	Z1307

^a^F, forward; R, reverse.

^b^All reactions had an initial denaturation at 94°C for 4 min and a final extension at 72°C for 4 min.

### DNA Isolation

*E. coli *isolates were obtained from a collection of strains stored in 15% glycerol at -80°C. Isolates were incubated at 37°C on LB agar plates overnight. Single colonies were picked and inoculated into 2 ml LB media and further incubated in a shaking incubator for 12-15 h at 37°C. A 1 ml suspension of bacteria was centrifuged, and DNA was extracted from the bacterial pellet with the QIAGEN DNeasy blood and tissue kit (QIAGEN, Valencia, CA).

### PCR

Amplifications were carried out in a total volume of 50 μl with 2 μl of template DNA (50-100 ng), 1 μl of each 20 mM primer, 5 μl 10× Accuprime PCR buffer II (Invitrogen) and 1 μl of Accuprime Taq DNA Polymerase (Invitrogen). PCR was performed in a GeneAmp PCR System 9700 (Applied Biosystems, USA). Primers were synthesized by Integrated DNA Technologies, Inc. (Coralville, Iowa). PCR conditions depended on the different target genes and primer sequences used are depicted in Table 4.

### Sequencing

PCR products were purified for sequencing with the QIAGEN QIAquick PCR purification kit. Both the forward and reverse strands were sequenced with the PCR primer set. Sequencing was performed at Cornell University Life Sciences Core Laboratories Centre using the Applied Biosystems automated 3730 DNA analyzer using big dye terminator chemistry and AmpliTaq-FS DNA polymerase.

### Phylogenetic analysis

Following sequencing of forward and reverse strands, the sequences were edited and aligned using Bioedit version 4.8.10[30] and converted into FASTA files. For each gene fragment, distinct alleles were identified and numbered by using the non-redundant databases (NRDB) program http://pubmlst.org/analysis/. When combined, the allele numbers assigned to each of the nine loci constituted a strain's allelic profile or sequence type (ST). Thus, each distinct allelic profile was considered a unique sequence type. A dendrogram (based on Neighbor Joining Method) was constructed by using the web-based data analysis tool "Tree drawing" http://pubmlst.org/analysis/ which uses the PHYLIP suite of programmes to generate neighbor-joining and UPGMA trees from allelic profile data. eBurst, a noncommercial algorithm previously designed for MLST of bacterial pathogens, was used to divide the 33 *E. coli *isolates into clusters of genetically related strains. The eBurst algorithm groups strains according to their allelic profiles by employing a user-specified group definition, which is the number of alleles that the isolates need to have in common to belong to the same group http://www.mlst.net/. The relatedness among the 33 *E. coli *isolates was also assessed by SplitsTree analysis[31], an alternative algorithm for the analysis and visualization of evolutionary data that is not always best represented by a standard tree.

### Nucleotide sequence accession numbers

The nucleotide sequences obtained by sequencing of the PCR products of each allele of all genes have been entered into the GenBank databases under different accession numbers which are shown in Table 5.

**Table 5:** Nucleotide sequence accession number

Allele	Accession number
*arcA*	FJ187757 to FJ187789
*aroE*	FJ217401 to FJ217433
*dnaE*	FJ217434 to FJ217466
*espA*	FJ217467 to FJ217499
*gapA*	FJ217500 to FJ217532
*gnd*	FJ217533 to FJ217565
*mdh*	FJ217566 to FJ217598
*ompA*	FJ217599 to FJ217631
*pgm*	FJ217632 to FJ217664

## Competing interests

The authors declare that they have no competing interests.

## Authors' contributions

SR and YFC conceived the study, experimental design and prepared the manuscript. SR was involved in all stages of the experiment. JS assisted in sequence data analysis and phylogeny estimation. DLG, KAM and BLA contributed *E. coli *strains. All authors read and approved the final manuscript.
